# Prevalence and characteristics of myocardial crypts in Japanese patients referred for cardiovascular magnetic resonance

**DOI:** 10.1186/1532-429X-17-S1-P333

**Published:** 2015-02-03

**Authors:** Eri Watanabe, Yufuko Takahashi, Etsuko Fujita, Makiko Kimura, Haruki Sekiguchi, Fujio Tatsumi, Tsuyoshi Shiga, Ken Shimamoto, Masatoshi Kawana

**Affiliations:** 1 Cardiology, Aoyama Hospital Tokyo Women's Medical University, Tokyo, Japan; 2 Cardiology, Institute of Geriatrics, Tokyo Women's Medical University, Tokyo, Japan; 3 Cardiology, Tokyo Women's Medical University, Tokyo, Japan

## Background

Myocardial crypts are narrow, blood-filled invaginations within the left ventricle (LV) wall and detected by cardiovascular magnetic resonance (CMR). Recent studies show that a higher prevalence of crypts in patients with hypertrophic cardiomyopathy (HCM) and genotype positive but phenotype negative relatives. Other studies show that myocardial crypts are relatively common in the normal population and incidental variants of local myocardial structures. However, these studies were performed in the western countries and the prevalence and kinds of HCM are different between Japanese and Western people. We aimed to investigate the prevalence and characteristics of myocardial crypts in Japanese people by using CMR.

## Methods

We examined retrospectively 266 consecutive patients (mean age 63.0±15.4 years, 65.8% male) referred for CMR. Crypts were defined as >50% invagination into normal myocardium. We performed 1.5T cardiac MRI including conventional cine imaging and late gadolinium enhancement (LGE) imaging. The location and the number of crypts were evaluated by using a 17-segment model. The prevalence of crypts was compared between patients groups. We also investigated the location of LGE, family history and the first recognition of abnormal ECG in the patients with crypts.

## Results

Crypts were identified in 12 patients (4.5%). Among them, ten patients were with HCM (83%) and 1 patient with congenital heart disease and 1 patient with arrhythmia but with normal structural heart. The prevalence of myocardial crypt is significantly higher in patients with HCM (10 of 82: 12.2%) than in patients without HCM (2 of 184: 1.1%) (P=0.0002). In 10 patients with HCM and crypt, 3 patients had apical hypertrophy. There was no significant difference in prevalence of crypts between HCM patients with apical hypertrophy and those without apical hypertrophy (3 of 23:13.0%, 7 of 59: 11.8%, respectively). Eight patients had single crypt and 4 patients had multiple crypts. All patients had at least one crypt at LV basal level and most popular location was LV basal inferior wall (10 patients). LGE was found in 6 patients and all were with HCM. Two patients had LGE in the same segment with crypt. There were 5 patients with HCM with family histories of sudden death or LV hypertrophy or dilated cardiomyopathy, but no patient without HCM had family history. Abnormal ECG findings were detected under age of 20 in four patients with HCM but in no patients without HCM.

## Conclusions

Myocardial crypts were more frequently seen in the patients with HCM and also seen in the patients with other cardiac diseases in Japan. Although genetic tests were not performed in these patients, genetic factors were suggested in the patients with myocardial crypts and HCM.

## Funding

N/A.

